# Negotiating adolescents' physically active life during the school day

**DOI:** 10.3389/fspor.2025.1505189

**Published:** 2025-03-07

**Authors:** Sara Hoy, Britta Thedin Jakobsson, Carolina Lunde, Håkan Larsson

**Affiliations:** ^1^ Department of Movement, Culture and Society, The Swedish School of Sport and Health Sciences (GIH), Stockholm, Sweden; ^2^ Department of Psychology, University of Gothenburg, Gothenburg, Sweden

**Keywords:** agency, health promotion, physical activity, whole school approach, school context, health equity, youth, ethnography

## Abstract

**Introduction:**

School contexts are addressed as important for encouraging adolescents' physically active lives, where whole-school approaches have emerged as globally recognized strategies. Recent research emphasizes the need to further understand the contexts relating to physical activity (PA) and strategies to enhance students' and staff's agency in relation to PA opportunities. In the current study, we explore early adolescent students' daily PA from an ecological perspective, examining the negotiated opportunities and barriers to PA within differing school contexts and how individual agency is expressed in relation to PA.

**Methods:**

This ethnographic collective case study was conducted in four Swedish middle schools that varied in size, resource denseness, and whether they were independent or public providers. The main empirical material was collected through ∼720 h of fieldwork during a school year, along with 86 interviews involving 50 students and 52 staff members. A comparative reflexive thematic analytical approach was used.

**Results and discussion:**

The transition between educational stages brought changes that influenced students and staff's agency related to PA. Against this backdrop, the analytical findings were organized into four themes. Students' PA was negotiated against the logic associated with being a “good” middle school student and teacher. Realizing daily PA also stood in relation to an anything-is-possible spirit, which was pitted against the lack of an organizational structure and high hopes for PA outcomes—creating a tension between vision and practicality, where student voices were overlooked. While all four schools claimed a commitment to providing PA opportunities for all, students negotiated their agency based on gender, age, social status, and previous experiences with traditional sports, which dominated recess activities. Students' PA during and after school was closely interconnected, especially expressed in physical education and health classes. This connection often benefited already active students in resource-rich environments while marginalizing those who were less active, further creating an uneven playing field regarding PA opportunities. Various schools shared challenges connected to students' daily PA, but challenges differed between and within schools. Future school policies, practices, and research should aim at addressing cultural, structural, and material dimensions focusing on sustainability, equity, and pedagogical issues, enabling young people to develop autonomy and ability to shape their PA experiences in ways that are meaningful to them.

## 1 Background

School contexts are often addressed as important settings for encouraging adolescents' physically active lifestyles and addressing the decline in health-promoting physical activity (PA) levels that usually occurs during early adolescence (1). Whole-school approaches have emerged as globally recognized strategies for promoting supportive school environments (2–4). These approaches are essential components of broader, systems-based initiatives designed to foster meaningful experiences that encourage a lifelong relationship with PA by providing multiple opportunities for daily activity (5–7). However, these initiatives also come with challenges. Several scholars have recently called for a rethinking of schools as a setting for PA promotion (8, 9). There is a need to understand the contextual environment of school systems by promoting qualitative and case study research agendas beyond positivist perspectives (8). For school-based PA, Jago et al. (9) suggested that key factors include the school setting; the demographic profile of the students and their interests; the availability of facilities; the attitudes, training, and skills of school staff; and school priorities. Importantly, future research should explore ways to enhance students' and key stakeholders’ agency with regard to PA, as well as what and how this agency is facilitated or hindered across differing school contexts (10). When considering people and their contexts, an essential starting point is the perspectives adopted. Recent research advocates for a more dialectical (relational) viewpoint that highlights the mutual influence between actors and their environment, as well as the agency that emerges from this relationship (10–12). In other words, people and their environments are interconnected; neither exists independently. This conceptualization of agency underscores the importance of both individual capacities and contextual dimensions and forms this article's ecological point of departure (13, 14). In light of these future directions, the current study explores how agency—a person's capacity to act based on their own and surrounding conditions (14)—is realized among actors in school contexts connected to students' daily PA through an ethnographic comparative collective case study conducted in four Swedish middle schools.

Schools have the function to juggle many roles connected to educational and caretaking missions, with fostering student agency in health and well-being being just one of many mission. The Ottawa Charter for Health Promotion served as a foundation for health-promoting school approaches that highlighted salutogenic perspectives on student health (15). Since then, many school initiatives have targeted students' PA as a health behavior, yet their success have been limited (1). Policies that support adolescents' daily PA in schools may be promising, yet current knowledge cautions against a “one-size-fits-all” approach and emphasizes the need for further investigation into how these policies translate into practice (16). Recent research on “best practices” for whole-school PA programs identified three key elements across various school contexts: an established school-based leader, support from the school community, and a wide range of available PA opportunities (17). However, the authors also stress the need for further investigation to better understand how these practices contribute to active school cultures. In addition, exploring the diversity within and between schools is important (18), as it highlights issues of inequality/equity, which are continuously reported in PA research within school settings (19). School settings can sediment and even increase inequalities through structures, cultural norms, and personal dispositions connected to PA—something that needs to be acknowledged when addressing the (re)production of PA inequalities (12, 20). Ethnographic methods are explicitly designed for studying cultures, with great potential to incorporate a whole-school approach within their methodologies. These methods are also advocated for in connection to implementation research within educational contexts (21). Yet, little ethnographic data seems to exist on how schools struggle with meeting the task of implementing and disseminating PA initiatives during the school day, as well as how these initiatives are negotiated by students and school staff.

### 1.1 The Swedish context

The Public Health Agency of Sweden (22) advocates for schools being key arenas for young people's PA practices, highlighting the need for greater attention in this area than it currently gets. Similar to international findings on declining PA among adolescents, Swedish research also indicates that aspects like gender, socioeconomic status, cultural background, and home environments influence activity levels (23–26). For example, differences were shown between girls' and boys' levels of PA during school hours but not during leisure time (27). These findings suggest that Swedish schools do not fulfill the compensatory role they are often claimed to constitute and illustrate both that PA initiatives appear to have limited ability to promote PA among the least active students and that these differences are allocated to school hours. Additionally, Farias et al. (20) showed how Swedish adolescents' experiences of a PA program differed in relation to their socioeconomic backgrounds, with their more or less busy schedule after school conditioning these experiences. Since Sweden has had many structural changes in the school system over the last decades, including the decentralization of management (28), it is important to point out recent research emphasizing the impact of local schools’ health and PA policies on adolescent students’s physical activity levels (27).

### 1.2 An ecological approach to context and agency

As mentioned previously, several scholars have highlighted the usefulness of the concept of agency in relation to adolescents' PA during the school day (10, 29). Similarly, teacher agency as an indispensable element in good and meaningful education has promoted the inclusion of this context-actor (relational) perspective in research related to school staff (30, 31). This agentic perspective focuses on increasing an actors' (e.g., student or staff) ability and space to maneuver or enact in a certain form of practice (e.g., PA) within a certain structure (e.g., school) through ongoing negotiations. Here, we use an ecological approach as our theoretical lens of exploration, drawing inspiration from the work of Priestley et al. (14). This ecological approach rests heavily on the conceptualization of agency, which emphasizes the importance of both individual capacities and contextual dimensions (13). First, agency is considered an emergent phenomenon that is “achieved by individuals, through the interplay of personal capacities and the resources, affordances and constraints of the environment by means of which individuals act” (14). Second, agency does not come from anywhere; instead, it is rooted in past experiences (iterational aspects) and orientated toward future short-term and long-term objectives, values, and aspirations (projective aspects). Finally, agency is enacted in concrete present moments (practical-evaluative aspects) (14). From this perspective, agency holds aspects that are both relational and temporal—being largely about negotiating the possibilities for different forms of (PA) actions available at particular points in time within specific contexts.

## 2 Aim

The aim of this ethnographic study is to explore students' PA during the school day across four different Swedish middle school contexts using a whole-school approach and an ecological lens of investigation. The analysis is constituted in the theoretical concept of *agency*, examining how daily PA is engaged in and perceived as possible by actors within and across these school settings.

The study is guided by the following research questions: *What are the opportunities and barriers for students' PA during the school day, and how are these being negotiated by students and school staff? How is agency expressed in relation to students' daily PA?*

## 3 Methodology and empirical material

This is an ethnographic collective case study embedded in an interpretive research paradigm and follows a short-term ethnography design described by Pink and Morgan (32). Our design included shorter periods of intense fieldwork of approximately 2 weeks at a time, conducted at three time points over the course of a year; the fieldwork was performed by a team of four researchers. The collective design encompasses what Hodge and Sharp (33) described as a combination of an intrinsic and instrumental case study approach, where we aimed to get both an in-depth understanding of the individual school contexts and these individual cases providing insights into the broader topic of PA during the school day. The study was performed in four middle schools located in the area of a larger city in Sweden: East Bridge School, Lakeview Academy, Southside Academy, and Springfield School. These schools vary in size, resource denseness, and management structure, operating either as independent or public institutions. The schools involved in our study were chosen based on a combination of convenience and strategic selection (socioeconomic variation in the catchment area and public and independent providers).

### 3.1 Generation of our data

The ethnographic methodology aims to describe culture(s), where researchers spend time in everyday contexts, engaging with fundamental aspects of human experience, such as cultural behavior, tacit and explicit cultural knowledge, and cultural artifacts (34). The first author (S.H.) conducted ethnographic fieldwork at two of the schools, while the second author (B.T.J.) was at the third school, and the third and last authors (C.L. and H.L.) divided their time at the fourth school. The fieldwork mainly involved participant observations, informal conversations, and semi-structured interviews. The participant observations varied in degree of participation from the researchers (from passive to active) and took part in areas within the different school environments, such as the schoolyard and proximal neighboring areas, and inside the school building itself. We visited classrooms, corridors, staff rooms, student leisure and club rooms, cafeterias, and all other spaces within the school setting. More importantly, we tried to engage with what people did, what people knew, and what people made use of within these spaces, where our exploration was guided by the intention to study how agency was shaped by opportunities and barriers influencing young people's physically active life during the school day. Different strategies were used to gain access and connect with different gatekeepers in the school settings. For example, spending time in the staff room helped us build relationships with many of the teachers and school staff in ways they did not feel watched or studied. It was more challenging in classroom situations, where it was obvious that we did not have a role other than observing those in the room. However, with time, most people within the schools became comfortable with us spending time there, even in classroom situations. Other ways of breaking the ice with staff and students included having lunch together or joining in on daily activities such as spending social time together between classroom activities. Doing sports or other PAs also provided a natural way to interact with students.

Approximately 100 h of fieldwork was conducted per school during the fall/winter of 2021, with an additional 80 h per school in the spring of 2022. Field notes were generated throughout the study period based on observations. If notes could not be taken at the actual observation, these were generated as close in time as possible to the moment of observation. To share our experiences with the research team, we held re-occurring meetings after every 2 weeks of fieldwork at individual schools. In addition, photos, drawings of school buildings, schedules, and other artifacts of the school environment were documented, primarily to create a shared understanding of each school within the research team.

Purposeful sampling was used throughout the study period to recruit participants for the semi-structured interviews who we believed could provide in-depth and detailed experiences related to our research aim. Participants included school management, staff responsible mainly for middle school students, staff and teachers involved in student health and/or PA, physical education and health (PEH) teachers, and middle school students (mainly 13–14 years old), ensuring variation in gender and PA engagement. During the interviews, we asked respondents about their background (e.g., staff members' professional roles and education, students' interests and family life), how PA was perceived and practiced at the school, and the possibilities and barriers associated with PA. In total, we conducted 86 interviews with 102 respondents. The characteristics of our data are presented in Table 1. Most of the interviews were conducted individually, while a few were done in small group constellations of two or three respondents. The duration of the interviews ranged between 9 and 58 min, with longer interviews typically with staff and shorter ones with students. Three additional interviews with students were conducted, but the students did not want these recorded. Therefore, notes from these interviews were treated as field notes.

**Table 1:** Data characteristics and number of respondents across the four schools.

Empirical material	East Bridge School	Lakeview Academy	Southside Academy	Springfield School	All
Fieldwork (h)	**180**	**180**	**180**	**180**	**720**
Fall/Winter 2021	100	100	100	100	400
Spring 2022	80	80	80	80	320
Interviews (*n* respondents)	**21 (24)**	**26 (32)**	**18 (25)**	**21 (21)**	**86**
Students (*n*)	**12**	**18**	**12**	**8**	**50**
Girls	6	9	8	5	28
Boys	6	9	4	3	22
School staff (*n* respondents)	**12**	**14**	**13**	**13**	**52**
Management	2	2	2	2	8
Student health team	2	4	2	2	10
Teachers	8	8	4	6	26
Of which are PE teachers	2	2	2	3	9
Other staff	–	–	5	3	8

Numbers in bold are total numbers for the organizing categories.

The interviews were transcribed verbatim by a third person through a transcription consultant company, with names and places anonymized in the transcription process. In addition, all transcripts were proofread and listened through for cross-checking against the audio recordings; this process also served as an initial step in the data analysis.

### 3.2 Ethical considerations

The four schools consented to participate in our study; however, the involved staff and students were not asked initially about the schools' participation as a whole. Because of the open-ended nature of ethnographic research, we adopted a situational ethical approach, as suggested by Hammersley and Atkinson (35), meaning that ethical considerations, such as respecting individuals’ integrity, confidentiality, and right to consent or disagreement, were judgments made in the context. Since our research involved young people aged 12–16 years, who are considered a potentially vulnerable group, ethical considerations were of high importance (36). One important ethical consideration in relation to our design and setting was the role of power structures at play. The four researchers were all of varying ages (35–61 years at the time) with ethnically Swedish appearances, were invested in topics related to PA and movement culture, and were affiliated with a higher educational institution. These played out in mainly two ways. First, as adults in a school setting, we were similar to school staff, where prevailing norms and structures carry a hierarchical power structure where young people are placed in a subordinate position (37, 38). Second, PA could be considered a “desired” and normative practice; therefore, it may have influenced how people in the fields interacted with us and around us as researchers of this specific topic. Ethical considerations did not end with “leaving the field”; rather, they remained a continuous process throughout the analysis and writing of this article. Since our empirical material was derived from interactions within environments that were partly culturally different from what we as researchers came from and involved vulnerable groups, we believed that it was important to maintain sensitivity in how we analyzed these data and portrayed our results and conclusions. For example, we have contemplated ethical considerations concerning the importance of conducting research in contrasting settings to ensure that voices of more vulnerable groups were heard, as well as reflected upon the concept of “othering” (39) to avoid stereotyping, stigmatizing, and moralizing vulnerable groups and topics.

All interviewees formally consented to participate by signing consent forms, which provided information about the study, its aims, and the voluntary nature of participation. Parents also had to consent for students to participate. The study was approved by the Swedish Ethical Review Authority (Dnr 2021-03834). To further protect the individuals who participated, we used pseudonyms for both schools and respondents and omitted their detailed descriptions throughout the material. There are at least two respondents in every category described for the formal interviews (see Table 1) to keep the confidentiality of the individuals themselves.

### 3.3 Analytical engagement with the empirical material

All phases of analysis were performed using the reflexive thematic analysis (RTA) method described by Braun and Clarke (40, 41). We chose RTA as our analytic approach derived for pragmatic reasons, given that our research team comprised several individuals collecting data at individual schools, each with different backgrounds in sports science, such as public health science and educational science. RTA offered us an accessible shared understanding of the analytic process, enabling us to identify patterns of meaning across the material through an interpretative approach.

The comparative approach in our study design was mainly related to the sampling and analytical process, drawing on the work of Bartlett and Vavrus (42) and their described “three axes” of comparison: horizontal, vertical, and transversal. In our study, we focused mainly on the horizontal and vertical aspects and compared both the commonalities and unique characteristics of the schools, creating “translocal” aspects of what was shared across cases and what differentiated them.

Our analysis started the day we stepped into the schools for our fieldwork and initiated the generation of data. As Spradley (34) pointed out, this way, we have had a cyclic approach to analysis rather than a linear one in our ethnographic work. Throughout our analysis, we have worked with the six phases of RTA suggested by Braun and Clarke (39, 41): (1) familiarizing ourselves with the data; (2) coding; (3) generating initial themes; (4) developing and reviewing themes; (5) refining, defining, and naming themes; and (6) writing up (this article). The research question regarding opportunities and barriers for students' daily PA (what) and the negotiations occurring in relation to those (how), along with the ecological theory and the concept of agency, guided the analysis. At each RTA phase, the analysis was performed according to the horizontal and vertical axes, where each school was analyzed individually and then compared to the other schools. This process was done with both field notes and interview transcripts, where initially we employed an inductive and semantic approach to the data, followed by a more deductive and latent approach. For coding and theme development, ATLAS.ti version 23 software was used.

As an example of our initial process, we highlighted sections in the material for each school that manifested as a barrier or opportunity (e.g., teachers perceiving there was no time for implementing PA for students in the classroom). Further, we looked for how this barrier/opportunity was related to other people or artifacts in the school context (e.g., if the teacher expressed the barrier of time in relation to something, for example, subject matter or a messy student group). We then coded the whole material in relation to the research questions using a similar approach to the initial process. This was also performed by analyzing each school by individually, followed by a comparison across schools. For example, a rather broad code group we created was “Students are active,” whose sub-codes were, e.g., “Wearing sports clothes at PEH,” “Boys play football, girls are on their phones,” “Students are active in the classroom if the teacher tells them to,” and “Physically activating students for study performance.” This code group exemplified a challenging case that required multiple reviews of the material and was eventually divided among themes, with sub-codes corresponding to different aspects of the ecological theoretical framework we used. In the second stage of analysis, we used our theoretical concepts and analyzed how this barrier/opportunity was enacted upon in the present moment (considering cultural, structural, and material aspects) and what past experiences and future objectives were in play. Overall, initial themes were generated by drawing a mind map of dominating meaning patterns from compiling codes into larger code groups or splitting them up while continuously revisiting the data material, earlier comments, and reflections on the material. The final themes were refined in relation to what was unique for the individual school contexts and what they shared as common patterns. An example of additional data excerpts connected to themes is provided in Table A (Supplementary Material).

Throughout the fieldwork and analysis process, meta-comments were written down by every individual researcher involved, which was further discussed with the whole research team to strengthen the study's credibility.

## 4 Ebb and flow: a common tale of a school day (descriptive findings)

The bright school corridor echoes empty, the classic metal lockers line the walls. At the far end are some benches and tables, also empty. Suddenly the bell rings and the murmur of the neighboring classrooms is clearly heard. A few seconds later, loads of students come tumbling out into the corridor. Some carry heavy books to the lockers; a few boys run to the material locker to get a soccer ball. Soccer, an activity that others go out of their way to avoid participating in. A group of girls retrieve their mobile phones from the teacher, because the policy says that if they go outside (and sit down) they are allowed to have them on their lunch break. A few students seem to continue their studies at the deployed tables and benches.

The vignette illustrates all four case schools—*East Bridge School*, *Lakeview Academy*, *Southside Academy*, and *Springfield School*. In this section, we provide a descriptive overview of these cases and illustrate what a typical school day may entail, which is followed by our analytical results in the subsequent section (Section 5).

**Figure 1:**
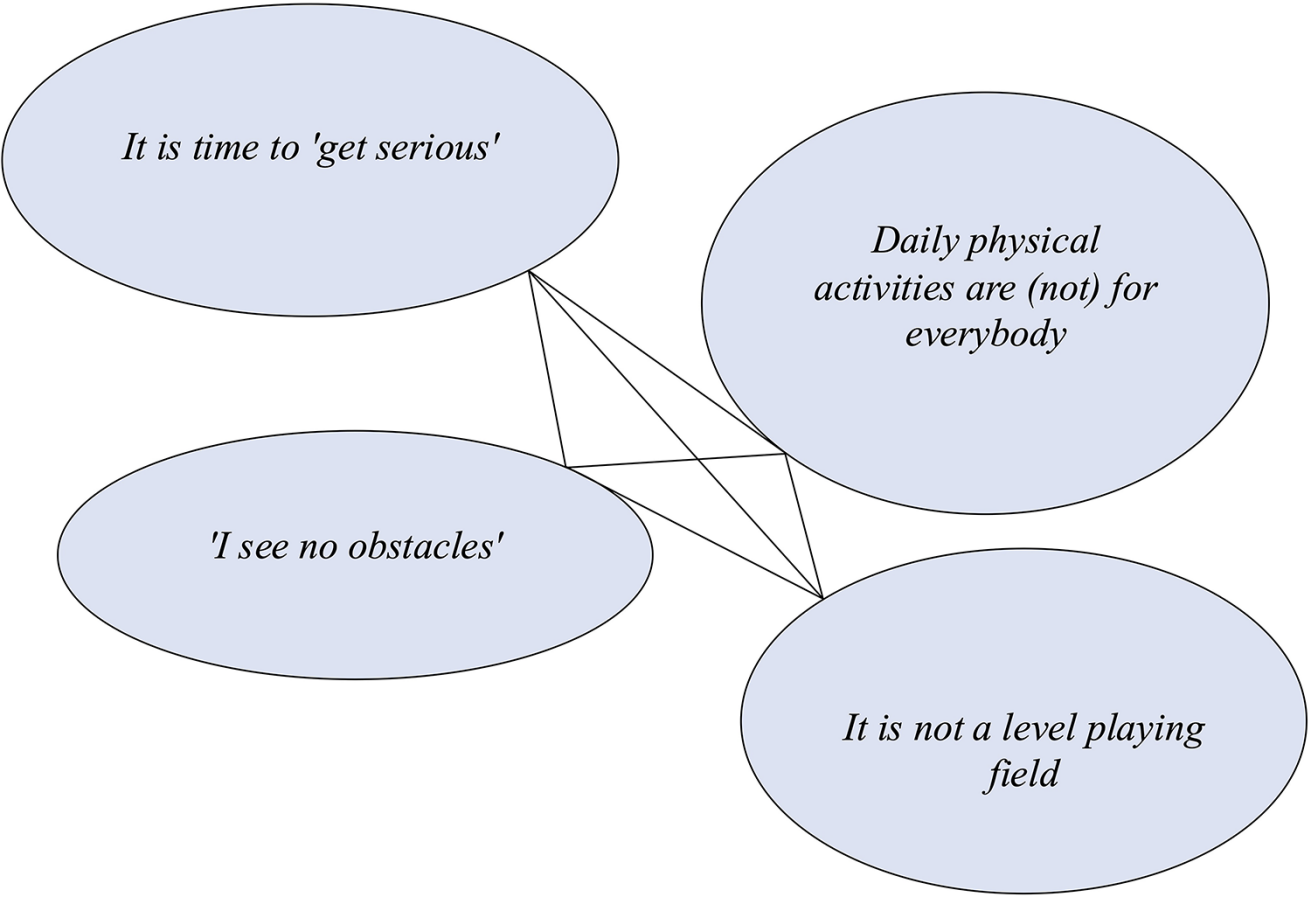
Characteristics of the four schools.

All four schools were located in suburban areas connected to a larger city in Sweden. All the schools had easy access to public transportation and were located in neighborhoods with residential areas, preschools, and local shops, including fast-food restaurants and grocery stores. One school was near a large shopping mall. The residential areas around the schools included various socioeconomic groups, with students coming from different catchment areas based on each school's profile. One of the schools was small, with fewer than 50 middle school students, while two of the schools had approximately 160 students; the largest school had approximately 600 students. Although the schools also offered elementary eduation, this aspect was excluded from our study. The individual characteristics of each school are displayed in Figure 1.

There were clear contrasts between movement and stillness during the school day, mainly in relation to the building blocks of the schedule: class and recess. Here, PA came in many forms and occurred mainly during practical-esthetic and academic classes (more or less), during recess, and along transport routes in the school premises, for example, between classes located in different parts of the school buildings. Not least, many students and school staff seemed to agree that the PEH subject, to some extent, fulfills the schools’s PA assignment as outlined in the general national curriculum (43). However, this was thought of and implemented to varying extents in the different schools, where two schools had PEH scheduled for middle school students three times per week, while the other two schools had it scheduled two times per week. Two schools had their own gym halls, while the other two schools rented a hall, one located very closely to the school and the other requiring approx. a 10-min walk for students and staff. The possibilities for PA also differed across all four schools depending on the physical school buildings and premises. A common feature during recess was outdoor access for mainly soccer, basketball, king (a ball game involving a basketball in a drawn square on asphalt played by four players), and sometimes ping pong/table tennis (some had this indoors), along with a few other alternatives. However, some schools had soccer fields located on the school premises, while others had access to one in the nearby area outside of the schoolyard, which implicated the presence or absence of school staff. Weather also influenced whether the students even went out or not. In middle schools, new rules compared to elementary schools allowed students to stay indoors during recess (with or without access to mobile phones). The indoor areas varied across schools, but common indoor rules included no running allowed and preventing students from “messing around” at all costs. Therefore, the types of PA permitted indoors were mainly less intense, for example, transporting oneself between points A and B.

The transition from elementary to middle school brought both organizational changes and social changes. The characteristics of the school day transformed due to a subject-focused schedule, with different teachers coming and going throughout the day, each teaching their subject to the students. Compared to elementary school, where one or a few main teachers were responsible for the entire school day, this was a big change that came with increased student responsibility, new rules, and a lost perception of the students' whole day among the school staff. At the same time, there was more curricular subject matter to be taught during class for the teachers, which ultimately should result in the students getting passing grades—a goal with both short-term and long-term objectives. These organizational changes tightened the schedule, leaving less time between classes for teachers to build relations with students and for other less prioritized tasks, such as PA promotion. The social climate transition brought about tangible changes concerning social relationships, with increased self-awareness among students in relation to oneself and others. While this seemed similar across all four schools, it played out a little differently depending on “who” and “what” that was related to and varied between classes and groups.

Overall, PA was not followed up on or evaluated by the schools, except during a one-time student health interview in middle school (which included questions about the student's PA level, usually carried out by the school nurse), and the teaching in the PEH subject was assessed and graded. We also observed that the student health work had an explicitly individual focus, while the PEH subject had both individual and group focus. The schools' various measures to implement (more) PA during the day focused on a group level, where everyone was offered the same opportunities described earlier. However, these opportunities were utilized by different students to a more or lesser degree. Our observations of students' daily PA were further voiced by Gittan, a special education teacher at Springfield School, who shared her thoughts concerning schools' PA assignment:

Gittan: Um … Yes, but I understand it [the general PA curricular policy] well as it is stated, but then I think that the school is like, it is a…, colossus, a colossus that is difficult to move, so in order for something to work, like with movement and sports, it would have been really easy if it was just like, we take the students out for recess and then they run and play games. It's not like that. Those who do it on their own, they have physical activity in their spare time too, they are not difficult to get started [to move during school time]. Those who are difficult are those who don't like to move. (…) So then, the question is, how are we going to bring in movement as well as everything else that goes into this “pot” [the school]. (…) How are we going to get it [PA] implemented in a way that works, without taking the focus away from the schools' educational mission that we also have to do, and in a way that works, because it also needs adult resources. Hmm, I think that is the challenge, how do we get it [PA] “in” in a natural way that is possible to follow up, and to actually be carried out.

As Gittan illustrates, integrating PA into the school day posed a significant challenge for schools, which are complex traditional educational institutions. This difficulty was further compounded by the reciprocal relationships among people within these contexts. These actors interacted with each other and were closely tied to the sociocultural, material, and temporal elements of their school environments, highlighting the interconnectedness between the structural characteristics of the school context and the individuals within them (agency).

In relation to the presented school cases, our analytical findings highlight identified themes that reflect broader patterns of meaning both within and across the schools against the backdrop of the above-presented findings.

## 5 Negotiating students' daily physical activity (analytical findings)

**Figure 2:**
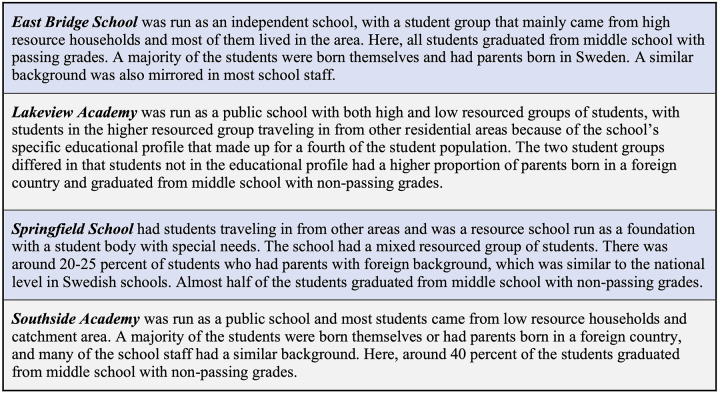
Analytical themes.

Our analysis displayed the negotiations concerning the facilitation and hindrances to PA in relation to our four school contexts, both within and between them. These negotiations were accomplished in connection with the perceptions and practice of students' physically active life during the school day and divided, yet interlaced, into four themes: *It is time to “get serious,” I see no obstacles*, *It is not a level playing field*, and *Daily physical activities are (not) for everybody* (see Figure 2).

### 5.1 It is time to “get serious”

“Have a seat, please!”, the teacher calls out. It is Thursday morning, at 9.55 am, and all the students in the classroom go from standing up next to their assigned seat to sitting down, ready to start today's school work. I have learned over the course of this fieldwork that this act, where the students stand up in the beginning of class until the teacher gives their permission for them to sit down, happens every lesson independent of teacher because it is part of how they “do it” at this school. All students are sitting by their desks, yet, one of the boys stands up again and goes back out to the corridor to get his books (he moves around and goes out several times during this lesson, by the way, for different reasons, e.g., bathroom visits and such). Now, everyone is sitting. Classic teacher-centered teaching. The teacher goes through what today's school work will consist of on the board (it is math class). After, students work independently sitting down, some students go and get computers for the calculator function, because there are not enough calculators. At 10.40 the classroom environment is getting noisy, and the teacher speaks up, telling the students to be orderly. He threatens to contact the parents, “this is a workplace, and I'm tired of having to speak up.” At 10.45, class is over, the teacher leaves while the students stay for the next lesson with another teacher teaching another subject. (Fieldnote)

The transition from elementary school to middle school and its changes meant that it was time to “get serious” and take personal responsibility in school, with grades and the (adult) future as long-term goals. It also meant that there was “no more room for play,” where PA was negotiated between being something serious or frivolous by both students and school staff in relation to being a middle school student and the educational mission the school had. Being a “good” teacher was to teach what was stated in the curriculum connected to each subject matter and ensure that all students met the knowledge requirements. For some teachers, PA in organized form became a valuable means of learning, in line with this ambition; for others, PA was seen as a non-serious endeavor that took time away from learning. For most, the idea of a “good” student implied sitting still and having full focus on the school task (if the movement was not part of the teacher’s instructions), where movement became an act resistance and non-movement signified docility. There was an overall sense of not having enough time to teach the curriculums' subject matter among teachers and the school management. Sture, one of the school managers at Lakeview Academy, described this when talking about the school's PA assignment: “Movement, yes, great but…, not at the expense of the protective factor that entering high school is for a child.” Here, the negotiation of PA was weighed against schools’ educational assignment to meet the requirements for the next level of education. Even though the objective of reaching these requirements was long-term goals, they became very real in the present moment of daily school life.

Many students across the schools expressed a desire for more movement during class; however, the objective of doing so often seemed connected to either taking a break or escaping school work. The movement did not seem to be the main goal, except for being a contrast to the sedentary school life. For some, more often boys, PA in the form of getting something from the locker, going to the bathroom, or just simply moving around in the classroom seemed to be a way to resist and negotiate the ideas of a sedentary student role. When talking to Georgina and Amelia, two students at Southside Academy, concerning the PA assignment that schools have, they said, “Physical activity, i.e., moving around? Then they [at the school] are lying. We sit in the classroom all the time (…) We go out if we have PEH class, in other lessons there is no movement.” When we continued our discussion, they described how they would want to move more during class: “5 min [of leg stretchers] is enough, to go outside, go to the toilet, get some fresh air, maybe drink water … Not very long. You want to learn something.” Making sure that you learn and being a good student was prioritized over moving/messing around, especially among girls.

The seriousness of PA as a means of learning and the negotiations connected to it varied across the different schools. When PA was implemented in the classroom context, it was negotiated in relation to whether the student group was messing around or not and what their study habits were. When teachers who implemented PA described how they judged whether it was appropriate or not to instruct students to move in the present moment, a compliant (studying) group of students (sitting down) was less challenging than a messy one (not studying, not sitting down). If the moment seemed appropriate, less intense versions of PA were implemented, with dancing to the instructions of YouTube being among the most intense examples. However, most teachers still did not incorporate movement in their classrooms, and when they did, the approach differed between schools. Lakeview Academy had a schedule where not many classes were longer than 60 min, so the judged need for breaking up those minutes did not seem to occur as often. Teachers and staff at Springfield School most often negotiated PA breaks based on individual student needs, often tied to their special needs, where techniques like walk-and-talk and other activities were used to offset an inter- or intra-relational conflict the student was experiencing within the school environment. At Southside Academy, the implementation of organized PA breaks did not seem to happen much at all, negotiated by staff in relation to that student groups had less habit of studying and many lessons often had a messy classroom environment. On the contrary, East Bridge School had more teachers incorporating these physical breaks. Here, the classroom environments were more often calm, with the majority of students having strong study habits and high levels of support from their families and the student health team for individual study arrangements; additionally, the school has a majority of students coming from homes where physical activities were highly valued. There were contextual differences across schools regarding study environments connected to PA, which gave teachers and other staff members varying space to maneuver in these negotiations.

The agency enacted in relation to this theme stood in close connection to the idea of being a good middle school student and a good teacher, with future objectives of short- and long-term goals—initially passing subject courses and ultimately finishing school with passing grades. This pattern was dominant across all four schools. However, differences emerged in the level of academic goals, with some schools aiming for excellence, while others focusing on merely achieving passing grades. Additionally, variations existed in student habits, with some groups demonstrating a strong past habit of studying and adhering to the role of a serious student, while others struggling to conform to these expectations. Other iterational aspects that influenced individual agency here were mainly related to school culture, with roles and expectations of what it meant to be a good middle school student and teacher, which involved students listening to the teacher, sitting still, and performing the task they had been given, as well as complying to ideas like no running in the corridors and such. Importantly, agency was enacted in the light of the cultural ideas of what was considered *being serious* with the educational endeavors of middle schools. PAs were enacted or implemented in relation to whether they were aligned with these dominating ideas. Structural and material aspects influenced individual agency. A clear example of this was found in the classroom, where the power dynamics of the teacher–student relationship were enacted through the arrangement of the room. The furniture layout suggested that teachers should stand at the front near the board, while students were expected to sit on their chairs and at their desks. However, many students across schools expressed in different ways their wish to move more in relation to these dominating logics, with PA becoming either something that would be enacted yet not take up much room or something that was an act of resistance to the sedentary culture of (serious) school work. In sum, the transition from primary to middle school meant it was time for students and staff to get serious, with (PA) agency negotiated against this discourse.

### 5.2 “I see no obstacles”

Richard: So, there are no obstacles to that [PA], I think. (…) But really, there are no such obstacles. Then…, it is not just that we must work outside the classroom, you can work in the classroom with physical activity too.

Researcher: Mm, yes, but, because you have discussed this a bit before, can you describe more how you think about it?

Richard: No, but more short breaks in, in the teaching with physical activity so that the students can get, yes, get the blood circulation going and start to “perk up” a bit, get moving, to get it as a natural part of the teaching.

Researcher: Yes. Do you think about this in all subjects or do you think about somewhere where it works better or worse?

Richard: No, it could work in all subjects. I see no obstacles.

One of the school managers, Richard, at Southside Academy stated that he “see[s] no obstacles” to the school implementing PA for students during both recess and class. This anything-is-possible spirit in relation to schools providing for student's daily PA was a common thread of thought among school management and staff. At the same time, the opportunities for PA were constantly negotiated in relation to the everyday challenges of the school context, where PA only could take 5 min from teaching time (to be practically possible) and needed to show immediate effects (to motivate staff to implement it). This way, there was chafing between the vision of PA's possibilities and the actual day-to-day possibilities of implementing it, where the visions clashed with practicalities of PA. The desired immediate effects sought were usually related to students' study focus and concentration, fewer conflicts, and better results in school assignments (for the future goal of higher grades described in the earlier theme). This combination of wanting an acute visible effect and the restricted space given to PA conditioned the anything-is-possible spirit, where PA was being put under high expectations of what it should do and function in a short amount of time.

Gittan, the previously quoted special education teacher, said, “Yes, there are a lot of possibilities, so, exactly, the challenge is the structure, I think. How can we achieve a [school] structure that is sustainable. Because we are the number of adults we are, and we can’t seem to add more tasks [for the staff].” As Gittan illustrated, there was an expressed lack of structure allocated to the PA assignment to make the visions of PA meet the practicalities of a school context. When being asked about what it would take for PA to be a more integrated part of the school day, Jasmyn, a special education teacher and part of the student health team at Southside Academy, said, “It takes…, structure, it takes stated people with stated responsibilities, which will lead this [PA assignment] all the time, who will come in during what periods of time, what is that person's purpose during that period.” This demonstrated what the structure would have to contain to make a difference, something many staff members valued but found challenging. The chafing between vision and practice filtered down to the lack of structure for addressing the PA assignment across schools. Several staff members expressed their concerns about adding PA to an already packed school day. In the absence of more integrated, concrete, and systematic ways of implementing PA—as well as the scarcity of having available personnel responsible for students' PA during the school day—a tension emerged between the anything-is-possible spirit, the pressed school structure with the schedule and resources, and the high hopes of what PA should do with its effects on the students. This tension was a dominant meaning pattern across the schools, where Ronaldo, a teacher at Lakeview Academy, illustrated how he negotiated his agency related to the issue of everyday practicalities as a teacher: “if you [the management] just say we should have physical activity, it is important that you do something for 5 min, I mean … I’m a math teacher, I’m not a movement teacher (…) I can invent stuff so, um, I don't mind but I, I'm not going to reinvent the wheel like, with the time I don't have.”

Overall, this theme mainly related to different roles of school staff and their concerns connected to schools' PA assignment, rather than concerning the students much. This was rather similar across schools. The lack of reflections on student involvement and the very few reflections from students themselves on the visions and practicalities of their daily PA also reflected *who* was perceived to enact this assignment. As Theodor and Alex, two 7th grade students at East Bridge School, recalled their experience of being invited to influence their PA possibilities at their schoolyard back in 3rd grade, they remarked, “It takes so much time for them [the school management]. There is a lot that they promise that doesn’t…, happen. We’re going to build a cable car, no that's not happening. Should we make swings, no it won’t happen. There will be a football field, no it hasn’t [happened].” They further explained how a few girls in their class who were student representatives kept on going to the school management with these wishes: “Yes, they really said, yes, yes, we’ll check, we’ll check. And since now 4 years later, nothing has happened.” Other examples of student involvement were mainly associated with student councils advocating for the addition of more breaks, shorter teacher-centered activities during class, or influencing the accessibility of sports equipment at recess (e.g., the number of basketballs). Importantly, adults were mainly illustrated to envision, plan, and implement PA. making it an integrated part of the school day through structured approaches. The role of adults vs. the non-participatory role students held displayed the (power) structures within schools, where adults mainly lead.

The anything-is-possible spirit in envisioning daily PA, combined with practical challenges (it can take 5 min) and high hopes (of immediate effects), was mainly tied to the lack of structured approaches to assessing the school's PA assignment. Nonetheless, our analysis suggested that past experiences and history influenced the distinct cultural roles of staff (adults) and students (children) in determining who was perceived to envision, plan, and implement daily PA for the students. This informed the agency among the schools’ actors, especially limiting student agency in the matter. The vision for more daily PA was related to future rather abstract ideas, yet the effects sought were related to short-term objectives—potentially difficult to attain and therefore challenging to practically evaluate in the present moment. Individual agency, as enacted in the present moment, was mainly related to the perceived lack and limiting aspects of the structure. Most staff members did not see themselves as responsible for enacting the PA assignment or having the capacity to do so. When students employed their agency in the existing structural work with PA, it was mostly related to material issues.

### 5.3 It is not a level playing field

It is right before lunch, and a light rain is hitting the ground on this fall day. This week I am back for field studies at one of the schools, and on my way to interview the PEH teachers. I am standing outside the sports hall, waiting to be let in, and literally cannot avoid the rain due to that there is no roof over the entrance to get cover from. I am thinking to myself what students might feel about waiting outside the sports hall when it is raining, and what that does to their experience of PEH classes. I also cannot help myself from thinking back on another visit at one of the other schools, and how their sports hall had both a warm glazed entrance, clean changing rooms, and big well-equipped premises. Suddenly, I hear the door being opened from the inside, and one of the PEH teachers lets me in. We walk through the changing rooms with graffiti on the walls and toilet paper along the floor on the opposite side of the room. The PEH teachers want to do our interview in the sports hall, and not their usual office. They are having issues with mold, and does not want to expose me (as they explain) to that situation (with me being visibly pregnant). (Fieldnote)

As illustrated, schools displayed different resources in the forms of materials such as available equipment and sporting premises, social structures involving staff and organization, and cultural values placed on a physically active lifestyle. These differences between schools were most often clearly illustrated in PEH classes; therefore, we used them to illustrate this theme. Our analysis showed how negotiations among staff and students connected to PA and sports during the school day were closely linked to different attractive attributes and various financial possibilities, which were connected to both *students' leisure time and home environments* and *individual schools' resources*. Students' physical experiences, aped by their (physically active or non-active) life stories, did not disappear upon entering school; instead, students carried these embodied past experiences and capacities throughout their daily school lives, where negotiations about PA opportunities took place. For example, there were differences between schools in how students had leisure PA or were members of local sports clubs. One extreme example was a school where the majority of students were members in some form of a sports or activity club, while another school had the opposite trend.

While joining in on some of the PEH classes and sports/field days organized at one of the schools, it was impossible not to notice the presence of local sports clubs, evident in the jerseys many students wore. A seemingly high proportion of students wore green shirts representing the local soccer club, with the majority being boys. This experience contrasted with PEH classes at other schools, where no students wore clothing representing sports clubs or sports/fitness brands other than the ordinary clothes they wore throughout the school day. PEH class, as both an illustrative and practical example, became a place where highly resourced students and schools and already sport-active cultures, or the opposite, negotiate PA in relation to these different logics. For example, PEH teachers could expect varying conditions regarding the equipment they had access to, both in terms of what was available in the premises and gym halls and what they could ask students to bring from home, such as skates, swimwear, shoes, sportswear, and other gear. The physical surfaces and premises offering opportunities for movement differed between schools. Schools had variable access to sports halls, and the condition of the premises also played a role. As the earlier illustrative example from our field notes, the symbolic value of a small and worn sports hall with mold problems stood in contrast to a large, fresh, well-equipped sports hall, where some students and staff members felt invested in school's PA assignment, while others did not.

Lakeview Academy, with two educational profiles—one with the characteristics of high socioeconomic status and one with contrasting characteristics—had negotiated their way to blend classes during PEH. This was due to students coming from different home environments, where some engaged in lots of sports activities while others did not. As stated by the management and the PEH teachers, the objective behind blending classes at PEH was for students “to share [values, embodied physical capacities and] experiences with each other.” The experiences they referred to were, however, mainly connected to students' lives outside of school. In contrast, at Springfield School, they focused on adapting their PEH environment by emphasizing clearer rules for PA and how to practice social interaction and teamwork within their student groups. PEH classes and other opportunities for PA were designed considering earlier experiences of student groups at this school with sports and similar activities outside of school. Macce, a student at Springfield School, described a common dilemma faced by his student group (students with special needs), noting that their embodied narratives of failure and negative experiences related to sports were constantly being negotiated in everyday school life and PEH despite often originating from outside the school environment:

Macce: When I went, when I started soccer, I was maybe in the 2nd–3rd grade. But I've been playing hockey until, well, it could have been up to the 5th grade, maybe. I've played hockey my whole life.

Researcher: Mmm.

Macce: I was often on skates since I was little.

Researcher: So, and you stopped with that?

Macce: Yes, but around like 5th grade.

Researcher: Mmm.

Macce: Because the [others born] 2007 then, they, it was sort of together, so [the ones born] 2008–2007 were sort of together and in the end the [ones born] 2007 started being nasty to me.

Researcher: Mmm.

Macce: I don't know why, and then it made me stop.

Researcher: Mmm. So, you have quit because… No, but as you said, nothing felt fun and then…

Macce: And then they were shitty and called me names and stuff.

Macce’s illustration of earlier negative experiences connected to PA and sports was a story expressed by several students across schools. However, the negative experiences differed in what they related to. Some experiences were due to perceptions of being different and not coping with the social codes of sports teams or activities, while some were due to social structures where some students were excluded or socially punished.

The varying “starting points” highlighted the close connection between life during and after school in relation to PA. The cultural values, social structures, and material resources of both students and the school (with its staff and facilities) were particularly evident in PEH classes. While this pattern was consistent across schools, PEH classes showcased how these aspects were expressed differently from one school to another, showing that it was not a level playing field. Individual agency within these schools was constrained by existing contexts, where promoting a physically active lifestyle (in terms of values, capacities, and experiences) primarily benefited those who were already active, potentially marginalizing those who were less active. Although there were objectives aimed at fostering a physically active future, the focus was primarily on past experiences and the current school context, with short- or long-term goals appearing less pronounced. In conclusion, the diverse school environments presented varying “(dis)advantages” in promoting students' physically active lifestyles.

### 5.4 Daily physical activities are (not) for everybody

Yes, but in the school yard spontaneous sports, only boys feel like … it seems to be hard to break that [pattern]. It feels like they are very welcoming and say that the girls are welcome to join, but it doesn't feel like it's like that at all (…) like the girls just aren't interested. (…) I think there is a culture, a culture among the girls as well, that you should be a bit of this nail polish and lipstick and follow influencers and sort of keep doing things like that. And then it happens that…, they are afraid of looking a bit silly if they run around in sports clothes in the middle of the day, so it will endanger their make-up and their stuff like that. On the other hand, if they do it [sports and PA] as a thing in their spare time, in the evening, then it is a completely different matter because then, then it is part of the concept, then you have to wash off the make-up, put on sports clothes and then everything is serious and in order. It is not spontaneous. And then you get away with all that. (…) Maybe it is more common among boys to want to assert themselves in sports as well, to show their “best” in that way. That you get more status by doing so, putting a ball in the basket, out on the basketball court, that it is not as much status to get for a girl to do it somehow, among the other girls. You don't get rewarded as much, socially, I think.

Caesar, a teacher at Lakeview Academy, illustrated the (re)occurring negotiations among students regarding whether PA was socially rewarding. Schools structured their offered activities more or less according to this social landscape (through, e.g., age rules). The longer opportunities for students' PA during a school day included recess during lunch and PEH classes. Negotiations about who wanted and could use the physical indoor and outdoor spaces offered for PA were influenced by age, gender, social status, and previous experiences with sports and competitive elements. If access was negotiated to these spaces, there was often a continued negotiation regarding status and just wanting to be part of the PA (no matter what) in a tacit web of power relations.

Sports and PA were perceived as sensitive by many students, existing within the social landscape of middle school students with heightened self-awareness in their transition toward adulthood. These activities often came with the feeling of being exposed, closely related to (sporting or non-sporting) bodies and identities. When Benjamin, a student at Springfield School, who identified as someone who usually liked to move, was asked how he perceived the school's PA assignment, he responded, “That mission is good for the school and it's important for the students to move.” However, when asked about the barriers he was experiencing in connection to PA, he said, “I don't want to look bad in front of the other students, so I don't want to move …. It is embarrassing to do physical activity. It becomes bad to do activities. Better to just hang around and play cards. If I'm going to do something, I think I'll do it by myself [outside of school].”

In several schools, students had specific indoor and outdoor areas designated for their use, restricted by age. The availability of PA in these areas, combined with restrictions preventing middle school students from using facilities and equipment designated for elementary students, limited their opportunities for PA during longer recess periods. As a result, middle school students were discouraged from playing, even when they still desired to do so. As Yang, a 7th grade student at Lakeview Academy, said, “Outside here, the big one [school yard]. For middle school, I think…, it's a bit too, what should I say, because…, so for…, in elementary school there's this climbing frame and basketball court and such but there is, there are none of them for middle school students.” When he was asked if he wanted to have access to this, the answer was “Yes.” Age also became an important aspect in negotiating PA, where usually older middle school students gained access.

Another very clear dividing line across all four schools was gender, where girls' access was negotiated in relation to the schools' opportunities for PA, such as physical spaces, materials, and social norms. Highly resourced and already active students outside of school were prioritized in the school's physical spaces, especially boys with previous sports experience. Even if girls were active in sports, they did not have the same space for maneuvering. Leila, a student who was very active in soccer in her spare time, said, “Before you could also borrow, um, materials like from the class material cabinet, now you can … Or you can't, you can't do that (…) they are mostly occupied by the boys who go and play football.” During our talk, Leila also described how some students in her class, as well as in the parallel class, used to walk over to the soccer field across the bridge from the school to play during lunch recess. When asked if she also used to go, she said, “Me, I want to do it but it's usually like this, maybe not all my friends don't want to play with the boys, because all the boys are there. So that…, maybe they’ll play something that isn't a girl…, we want to play, some football game.” Another student, Amina, at Lakeview Academy, explained:

Researcher: Mm, okay. Have you been playing ping pong?

Amina: No. So, it's mostly like this, I don't know, so I like to play it, I usually do, we played it in PEH class but uh, I don't dare play with those [boys].

Researcher: Okay. What, are they scary?

Amina: No, no, so it's only boys like this from the 8th grade and some from my class, but I don't know, and there are usually never any girls playing. (…)

Researcher: Mmm. Why do you think there are so few girls?

Amina: I don't know. Most people tend to just hang out with each other instead of doing anything.

Even boys who were not portraying hegemonic masculine attributes—such as athleticism, toughness, or body size—negotiated these opportunities either by avoiding them or buying into the power plays that occurred, e.g., on the soccer field. One of the commonly referred power plays at East Bridge School was called “Butts Up.” Lucas, a student at East Bridge School, portrayed this play taking place when some of the boys lost a ball game during soccer:

Researcher: Yes. And when do you get “butts upped” then?

Lucas: When it is all over.

Researcher: Yes. Okay. How does it happen then?

Lucas: Everyone who has lost lines up and those who win get to shoot the ball as hard as they can [at the back bodies of the ones-who-lost]. (…) Yes, but it is a bit like a punishment.

The losing students were usually the same ones repeatedly, as described by both staff and students. When talking to Gabriel, one of the other students at East Bridge School, about how he would like to move more during a school day, he said, “Eh, I would probably play basketball, or maybe soccer.” When further asked if he could not already do that, with the basketball court within visual proximity during the talk, he continued, “Well, I can actually. But yes. It just doesn't work out that way. Because it's usually a little, it's usually the [other] boys who play basketball, so then we're not like jumping in, we're outsiders like this.” This highlighted the persistent boundaries through which students interact and the intersecting roles of various social positions within school settings, where different behaviors (such as PA) receive varying degrees of social reinforcement.

This theme—daily PAs were (not) for everybody—primarily pertained to the students within these school contexts but also included the physical environments provided and, to some extent, the organizational structure of the schools, such as the presence or absence of staff and the rules governing activities. Past life stories, such as sporting experiences and the historical and cultural meanings incorporated in physical premises, for example, a soccer field, conditioned staff and student's agency in negotiating students’ physically active lives during school days and their space for maneuvering. Future objectives related to students' agency in this theme were being socially rewarded, both in the short term and long term, such as being included in immediate events and social cliques, as well as forming lasting friends and social groups. The concrete, everyday contexts of schools' possibilities were conditioned by these cultural, material, and socially structured landscapes, illustrated above between schools and within each school.

## 6 Concluding discussion

In our ethnographic study, we explored negotiations of students' PA during the school day across four different school contexts and examined how agency was expressed in relation to this among students and staff—using an ecological theoretical framework and a comparative approach. The study shows how these negotiations unfold relative to iterational, practical-evaluative, and projective ecological dimensions (14). First, PA was negotiated against the backdrop of multidimensional transition challenges, including organizational structures and social changes from elementary to middle school, with transport routes, recess, and PEH perceived as means of “solving” the PA assignment. Against this, our study displays how PA-related agency was negotiated through the idea of being a “good” middle school student and teacher, with both short- and long-term objectives (projective) centered on passing grades and meeting knowledge requirements—where agency was expressed in relation to whether PA was considered “serious” within this educational endeavor in everyday moments (practical-evaluative). The realization of daily PA was also negotiated in relation to a sense of lack of structure (practical-evaluative), creating a tension between the vision of possibilities (primarily ascribed to staff, not involving the students) and the high expectations for immediate outcomes of PA initiatives (projective). Our findings further showed that students' physically active lives during and after school were closely interconnected, a connection particularly evident during PEH classes (practical-evaluative). This interconnectedness tended to benefit those who were already active in resource-dense environments after school, possibly marginalizing those who were less active (iterational). Students negotiated and expressed their agency in close connection to gender, age, social status, and previous experiences with traditional sports, which primarily comprised the PA options available during recess, such as soccer, basketball, and table tennis (iterational, practical-evaluative, and projective).

Our findings illustrated common meaning patterns across schools, yet these patterns were conveyed in varying ways in individual school settings. The complexities of the studied school settings displayed shared facilitators and barriers to students' daily PA and also revealed that these challenges varied between different schools and within the same school. In our comparative approach, we have focused mainly on horizontal and vertical comparisons of our four school cases, inspired by the work of Bartlett and Vavrus (42). The analytical findings highlighted that contextual challenges associated with schools' efforts to provide all students with daily PA were related to three interconnected structural layers: across, between, and within the settings. Here, we outline these layers as *translocal* (across schools), *interlocal* (between schools), and *intralocal* (within schools).

To exemplify the focal schools' *translocal* challenges, student agency connected to PA was limited due to the expectations of what a good middle school student should be like (sedentary and focused), yet this role was negotiated by students based on how they dealt with future educational objectives. Here, our results displayed that gender played a role, where girls had a narrower space to maneuver compared to boys, who more often used PA as a form of resistance against the sedentary student role, both in classrooms and other school premises. However, the constraints of agency regarding the idea of being a good student were also shown to differ *interlocally*, where some schools had a much higher proportion of students willing to conform to these ideas and objectives, usually with students coming from homes with stronger study habits. In their earlier research, Boonekamp et al. (44) performed a multiple case study in six Dutch middle schools, investigating how physical, social, and pedagogical contexts fostered students' agency in relation to PA. Like our study, their study also identified similarities and differences across schools in their efforts to facilitate student agency—highlighting a need across schools for pedagogical approaches that involve students' perspectives, participation, and reflections related to PA. However, this need appeared to be intertwined with the notion of how staff negotiated their willingness, competencies, and available resources in terms of support, vision, time, space, leadership, and the pedagogical models used (44). Combined with the current study's results, we suggest that pedagogical perspectives are important issues that future PA initiatives should address when aiming to enhance students’ and staff's agency. Similarly, our empirical findings suggest there is much room for improvement in developing sustainable long-term structural strategies containing salutogenic approaches and asset-based perspectives in national and local school PA promotion, as suggested by García Bengoechea et al. (8). Such initiatives could further enable young people to develop their autonomy and ability to shape PA according to what they value and find meaningful as part of their role as middles school students and becoming adults.

Another important finding of how individual student agency was constrained, both *translocally* and *interlocally,* was by the intersecting social power structures, sociocultural ideas, and economic and material resources connected to PEH classes and PA opportunities during recess. These issues combined created unequal playing fields, where inequalities risked being further sedimented. Earlier international reviews and meta-analyses support these findings, highlighting the need to address schools' activity cultures and inequalities that affect adolescents' physically active lives (45–47). In line with Garrett's (48) earlier findings, our study also illustrates how students, particularly young women and girls, navigated their physical identities, bodies, and behaviors within prevailing stereotypical gender discourses and power structures while engaging (or not) in PA. Furthermore, when the focal schools emphasized providing PA for *all*, it implied that students were granted equal access to hegemonic male forms of PA, typically defined by traditional sports facilities and competitive games. This dynamic likely reinforced gendered and socioeconomic inequalities in PA, as noted by various scholars in both international and Nordic contexts (12, 19, 20, 49). Just as Alliot et al. (50) pointed out in their systematic review, one student's barrier can be another student's facilitator, differing based on socioeconomic position at both group and individual levels. Especially, their results highlight how adolescents’ PA agency is facilitated or constrained by social support, access to physical spaces, gender, and narratives of health(y) behaviors, lining up with our findings.

Our schools exhibited varying degrees of local cultural homogeneity, particularly evident in *intralocal* contextual challenges. In all four schools included in this study, PA emerged as a relational phenomenon, influenced by interactions with other people and artifacts—highlighting the aspects of cultural nuances and social norms. For instance, when one student wished to play basketball, their ability to express that desire was constrained by the social status of the other boys in the group. In another example, a very active girl in her spare time was constrained to play soccer during school hours because of gendered norms. These findings again highlight inequity issues and support previous research by McMullen et al. (17), which indicates that offering a diverse range of PA opportunities is crucial for fostering an active culture. This implies that future school PA initiatives should address these inequities and their compensatory role, both locally and nationally. Co-creation and co-production processes could help PA initiatives better align with the ambition of a whole-school approach by involving both youth and staff. Such strategies are gaining interest and are advocated by researchers to include youth (51) and staff (52). However, Smith et al. (53) pointed out how co-production can be a challenging strategy driven by different reasons; they also suggested ways to co-produce “to advance the participatory turn in sport, exercise, and health research.”

Inspired by the work of Priestley et al. (14), our ecological approach has focused mainly on the inner school settings, examining the social, structural, and material aspects of contextual challenges that shaped how (PA) agency was enacted. During our study, additional outer setting factors such as political issues, leisure time, and home environments emerged. These factors were not captured in this article and could be explored in future research agendas. To add, we noticed that our presence as “PA researchers,” at least to some extent, influenced students and staff. Some staff members were willing to spontaneously talk about PA with us, and in spontaneous conversations with students, norms about PA emerged. However, we did not perceive that our presence changed the dominant patterns of PA and sedentary behavior within the schools. Even though we have considered these limitations, future initiatives could explore it further. A strength of the current study lies in its rigorous methodology and the size of its empirical material, involving differing school contexts, which aids in developing a rich understanding of the studied topic. However, the study is limited to a suburban Swedish context, which potentially limits the transferability/generalizability of its findings to other Nordic and international settings. The intention of the study was not to point out opportunities to generalize but to identify key questions that anyone who has the ambition to work on promoting PA in a school context needs to ask themselves, such as how the school contexts and social norms affect students' and staff's relationships with and approaches to PA.

To conclude, our findings display how students and staff negotiated agency connected to PA within intertwined ecological dimensions, where cultural, structural, and material aspects need to be further addressed in light of pedagogical, equity, and sustainability issues. The call for a “rethinking of PA in school contexts,” as suggested, for example, by Jago et al. (9), should not only consider the contextual challenges across and between schools but also further address local contexts and challenges within individual schools, adding relational/dialectic and salutogenic perspectives. This way, future strategies could be focusing on enhancing student agency connected to PA while acknowledging the intersecting socially and culturally coded power structures that shape students access to equal PA opportunities—accounting for the fact that PA opportunities truly are for everybody.

## Data Availability

The datasets presented in this article are not readily available because because ethical considerations connected to the school cases and the individual participants. Requests to access the datasets should be directed to sara.hoy@gih.se.

## References

[1] van Sluijs EMF Ekelund U Crochemore-Silva I Guthold R Ha A Lubans D Physical activity behaviours in adolescence: current evidence and opportunities for intervention. Lancet. (2021) 398(10298):429–42. 10.1016/S0140-6736(21)01259-9 34302767 PMC7612669

[2] Daly-Smith A Quarmby T Archbold VSJ Corrigan N Wilson D Resaland GK Using a multi-stakeholder experience-based design process to co-develop the creating active schools framework. Int J Behav Nutr Phys Act. (2020) 17(1):13. 10.1186/s12966-020-0917-z 32028968 PMC7006100

[3] McMullen J Ní Chróinín D Tammelin T Pogorzelska M van der Mars H. International approaches to whole-of-school physical activity promotion. Quest. (2015) 67(4):384–99. 10.1080/00336297.2015.1082920

[4] Milton K Cavill N Chalkley A Foster C Gomersall S Hagstromer M Eight investments that work for physical activity. J Phys Act Health. (2021) 18(6):625–30. 10.1123/jpah.2021-0112 33984836

[5] International Society for Physical Activity and Health, I. (2020). ISPAH’s Eight investments that work for physical activity. Available online at: https://ispah.org/resources (Accessed January 25, 2025).

[6] Rutter H Cavill N Bauman A Bull F. Systems approaches to global and national physical activity plans. Bull W H O. (2019) 97(2):162–5. 10.2471/BLT.18.220533 30728623 PMC6357559

[7] World Health Organisation. Global action plan on physical activity 2018–2030: more active people for a healthier world Geneva (2018).

[8] García Bengoechea E Woods CB Murtagh E Grady C Fabre N Lhuisset L Rethinking schools as a setting for physical activity promotion in the 21st century–a position paper of the working group of the 2PASS 4Health project. Quest. (2024):1–20. 10.1080/00336297.2024.2318772

[9] Jago R Salway R House D Beets M Lubans DR Woods C Rethinking children’s physical activity interventions at school: a new context-specific approach. Front Public Health. (2023) 11:1149883. 10.3389/fpubh.2023.1149883 37124783 PMC10133698

[10] Boonekamp GMM Jansen E O’Sullivan T Dierx JAJ Lindström B Pérez-Wilson P The need for adolescents’ agency in salutogenic approaches shaping physical activity in schools. Health Promot Int. (2022) 37(1):daab073. 10.1093/heapro/daab073 34142137 PMC8851412

[11] Spotswood F Vihalemm T Uibu M Korp L. Understanding whole school physical activity transition from a practice theory perspective. Health Educ. (2021) 121(5):523–39. 10.1108/HE-04-2021-0066

[12] Wiltshire G Lee J Williams O. Understanding the reproduction of health inequalities: physical activity, social class and Bourdieu’s habitus. Sport Educ Soc. (2019) 24(3):226–40. 10.1080/13573322.2017.1367657

[13] Emirbayer M Mische A. What is agency? Am J Sociol. (1998) 103(4):962–1023. 10.1086/231294

[14] Priestley M Biesta G Robinson S. Teacher Agency: An Ecological Approach. London: Bloomsbury Publishing (2015). Available online at: https://books.google.se/books?id=09hbCgAAQBAJ

[15] World Health Organization. Ottawa Charter (1986).

[16] Woods CB Volf K Kelly L Casey B Gelius P Messing S The evidence for the impact of policy on physical activity outcomes within the school setting: a systematic review. J Sport Health Sci. (2021) 10(3):263–76. 10.1016/j.jshs.2021.01.006 33482424 PMC8167338

[17] McMullen JM Kallio J Tammelin TH. Physical activity opportunities for secondary school students: international best practices for whole-of-school physical activity programs. Eur Phy Educ Rev. (2022) 28(4):890–905. 10.1177/1356336X221092281

[18] Keshavarz N Nutbeam D Rowling L Khavarpour F. Schools as social complex adaptive systems: a new way to understand the challenges of introducing the health promoting schools concept. Soc Sci Med. (2010) 70(10):1467–74. doi: 10.1016/j.socscimed.2010.01.034 20207059

[19] Guthold R Willumsen J Bull FC. What is driving gender inequalities in physical activity among adolescents? J Sport Health Sci. (2022) 11(4):424–6. doi: 10.1016/j.jshs.2022.02.003 35217213 PMC9338329

[20] Farias L Nyberg G Helgadóttir B Andermo S. Adolescents’ experiences of a school-based health promotion intervention in socioeconomically advantaged and disadvantaged areas in Sweden: a qualitative process evaluation study. BMC Public Health. (2023) 23(1):1631. 10.1186/s12889-023-16581-z 37626379 PMC10464358

[21] Moir T. Why is implementation science important for intervention design and evaluation within educational settings?. Front Educ. (2018) 3:61. 10.3389/feduc.2018.00061

[22] Public Health Agency of Sweden. (2021). Så kan verksamheter bidra till ökad fysisk aktivitet och minskat stillasittande: Ett stöd för nationellt, regionalt och lokalt arbete. Available online at: https://www.folkhalsomyndigheten.se/publicerat-material/publikationsarkiv/s/sa-kan-verksamheter-bidra-till-okad-fysisk-aktivitet-och-minskat-stillasittande/?pub=100393 (Accessed January 13, 2025).

[23] Fröberg A Jonsson L Berg C Lindgren E-C Korp P Lindwall M Effects of an empowerment-based health-promotion school intervention on physical activity and sedentary time among adolescents in a multicultural area. Int J Environ Res Public Health. (2018) 15(11):2542. 10.3390/ijerph15112542 30428548 PMC6267499

[24] Hoy S Larsson H Kjellenberg K Nyberg G Ekblom Ö Helgadóttir B. Gendered relations? Associations between Swedish parents, siblings, and adolescents’ time spent sedentary and physically active. Front Sports Act Living. (2024) 6:1236848. 10.3389/fspor.2024.1236848 38455967 PMC10918000

[25] Jonsson L Berg C Larsson C Korp P Lindgren E-C. Facilitators of physical activity: voices of adolescents in a disadvantaged community. Int J Environ Res Public Health. (2017) 14(8):839. 10.3390/ijerph14080839 28933747 PMC5580543

[26] Nyberg G Kjellenberg K Froberg A Lindroos AK. A national survey showed low levels of physical activity in a representative sample of Swedish adolescents. Acta Paediatr. (2020) 109(11):2342–53. 10.1111/apa.15251 32266736

[27] Nyberg G Ekblom Ö Kjellenberg K Wang R Larsson H Thedin Jakobsson B Associations between the school environment and physical activity pattern during school time in Swedish adolescents. Int J Environ Res Public Health. (2021) 18(19):10239. 10.3390/ijerph181910239 34639539 PMC8507782

[28] Ferry M Westerlund R. Professional networks, collegial support, and school leaders: how physical education teachers manage reality shock, marginalization, and isolation in a decentralized school system. Eur Phy Educ Rev. (2023) 29(1):74–90. 10.1177/1356336(221114531

[29] Kirk D Lamb CA Oliver KL Ewing-Day R Fleming C Loch A Balancing prescription with teacher and pupil agency: spaces for manoeuvre within a pedagogical model for working with adolescent girls. Curric J. (2018) 29(2):219–37. doi: 10.1080/09585176.2018.1449424

[30] Biesta G Priestley M Robinson S. The role of beliefs in teacher agency. Teachers Teach. (2015) 21(6):624–40. 10.1080/13540602.2015.1044325

[31] Priestley M Edwards R Priestley A Miller K. Teacher agency in curriculum making: agents of change and spaces for manoeuvre. Curric Inq. (2012) 42(2):191–214. 10.1111/j.1467-873X.2012.00588.x

[32] Pink S Morgan J. Short-term ethnography: intense routes to knowing. Symb Interact. (2013) 36(3):351–61. doi: 10.1002/symb.66

[33] Hodge K Sharp L-A. Case studies. In: Smith B, Sparkes AC, editors. Routledge Handbook of Qualitative Research in Sport and Exercise. London: Routledge (2016). p. 62–74. 10.4324/9781315762012.ch6

[34] Spradley JP. Participant Observation. Holt: Rinehart and Winston (1980). Available online at: https://books.google.se/books?id=sQClDJXc5vkC

[35] Hammersley M Atkinson P. Ethnography: Principles in Practice. London: Routledge (2007). Available online at: https://books.google.se/books?id=_fQnMAEACAAJ

[36] Quennerstedt A Harcourt D Sargeant J. Research ethic in research involving children: ethics as risk management and ethical research practice [Forskningsetik i forskning som involverar barn: etik som riskhantering och etik som forskningspraktik]. Nordic Stud Educ. (2014) 34:77–93. 10.18261/ISSN1891-5949-2014-02-02

[37] Darbyshire P MacDougall C Schiller W. Multiple methods in qualitative research with children: more insight or just more? Qual Res. (2005) 5(4):417–36. 10.1177/1468794105056921

[38] Gubby L. The importance of an organic process in ethnographic research: working with children in a physical activity setting. Phys Educ Sport Pedagogy. (2021) 28(2):109–20. 10.1080/17408989.2021.1955096

[39] Pickering M. Stereotyping. The Politics of Representation. Palgrave: Basingstoke (2001).

[40] Braun V Clarke V. Reflecting on reflexive thematic analysis. Qual Res Sport Exerc Health. (2019) 11(4):589–97. 10.1080/2159676X.2019.1628806

[41] Braun V Clarke V. Thematic Analysis: A Practical Guide. London: SAGE Publications (2021). Available online at: https://books.google.com/books?id=mToqEAAAQBAJ

[42] Bartlett L Vavrus F. Rethinking Case Study Research: A Comparative Approach. London: Taylor & Francis (2017).

[43] SNAE. Läroplan för Grundskolan, Förskoleklassen och Fritidshemmet 2011: Reviderad 2022. (9789138327500). Stockholm: The Swedish National Agency for Education (2022).

[44] Boonekamp GMM Dierx JAJ Jansen E. Shaping physical activity through facilitating student agency in secondary schools in The Netherlands. Int J Environ Res Public Health. (2022) 19(15):9028. 10.3390/ijerph19159028 35897397 PMC9331768

[45] Hartwig TB Sanders T Vasconcellos D Noetel M Parker PD Lubans DR School-based interventions modestly increase physical activity and cardiorespiratory fitness but are least effective for youth who need them most: an individual participant pooled analysis of 20 controlled trials. Br J Sports Med. (2021) 55(13):721. 10.1136/bjsports-2020-102740 33441332

[46] Love R Adams J van Sluijs EMF. Equity effects of children’s physical activity interventions: a systematic scoping review. Int J Behav Nutr Phys Act. (2017) 14(1):134. 10.1186/s12966-017-0586-8 28969638 PMC5625682

[47] Morton KL Atkin AJ Corder K Suhrcke M van Sluijs EMF. The school environment and adolescent physical activity and sedentary behaviour: a mixed-studies systematic review. Obes Rev. (2016) 17(2):142–58. doi: 10.1111/obr.12352 26680609 PMC4914929

[48] Garrett R. Negotiating a physical identity: girls, bodies and physical education. Sport Educ Soc. (2004) 9(2):223–37. 10.1080/1357332042000233958

[49] Jansson A Brun Sundblad G Lundvall S Norberg JR. Exploring the intersection between students’ gender and migration background in relation to the equality of outcome in physical education in Sweden. Sport Educ Soc. (2024) 29(1):42–57. 10.1080/13573322.2022.2110862

[50] Alliott O Ryan M Fairbrother H van Sluijs E. Do adolescents’ experiences of the barriers to and facilitators of physical activity differ by socioeconomic position? A systematic review of qualitative evidence. Obes Rev. (2022) 23(3):e13374. doi: 10.1111/obr.13374 34713548 PMC7613938

[51] Williams T Ward K Smith M. Conceptualization of co-creation, co-design and co-production with children for health-promoting physical environments: a systematic search and scoping review. Child Youth Environ. (2023) 33(2):1–38. 10.1353/cye.2023.a903096

[52] Alfrey L O'Connor J Jeanes R. Teachers as policy actors: co-creating and enacting critical inquiry in secondary health and physical education. Phys Educ Sport Pedagogy. (2017) 22(2):107–20. 10.1080/17408989.2015.1123237

[53] Smith B Williams O Bone L, Collective, The Moving Social Work Co-Production. Co-production: a resource to guide co-producing research in the sport, exercise, and health sciences. Qual Res Sport Exerc Health. (2023) 15(2):159–87. 10.1080/2159676X.2022.2052946

