# Supplementary material for: Negotiating adolescents' physically active life during the school day
Source: Front Sports Act Living. 2025 Mar 7;7:1505189. doi: 10.3389/fspor.2025.1505189 (PMC11925886; doi:10.3389/fspor.2025.1505189)
Supplement: Supplementary file 1 [file Datasheet1.pdf]

Table A. Examples of data excerpts from process for analytical themes

Quotation	Codes	Themes
Student: Outside here [the school yard], the big one. Middles schools' [yard], so I think it is a little too, what to say..., so the elementary school has this climbing station and basketball court and such, but there are none of those for us in secondary school.<br>Researcher: There is nothing?<br>Student: Yes, no.<br>Researcher: So, you would have liked to have this too?<br>Student: Yes.	Elementary school students get to climb and play (basketball)<br><br>Outdoor areas (school yard) are reserved for younger students<br><br>Middle school students are supposed to do other things than to move	*It is time to 'get serious'*
Researcher: I've seen some students who kind of hurry off and play ping-pong.<br>Student: Yes.<br>Researcher: During recess, and then they run back.<br>Student: But those ones [male students] usually come late, the ones in my class. They, well, what do you call it, if they keep track of the time, they won't do it [get late], even though they get so 'into the game'.	Some care more about sports than studying<br><br>Loosing track of time when moving/sporting<br><br>Being late for class (is okey for boys)	
Teacher 2: The football field is very far away. We usually play, used to, sometimes we play…<br>Teacher 1: Basketball.<br>Teacher 2: Basketball, because there is a basketball field next door so… We take that chance that the school has given us.<br>Teacher 1: So, we have quite short breaks, so…<br>Teacher 2: Almost none.<br>Teacher 1: It is very difficult to go out and play basketball like this, it is fun but you don't have time for it because you have to go to class and study and…	Middle school students have short breaks<br><br>Schools offer different sporting possibilities<br><br>Physical activity is not serious in relation to studies	

Researcher: Mm.<br><br>Teacher 2: And some students choose to study instead of having a break.		
Teacher: Because I mean, you as a class teacher in elementary school who thinks like – 'I take them [the students] for a walk now' – then I 'gain the time back' in the afternoon, then they have better concentration, they can sit still. If I, as a subject teacher in middle school, have them in the morning for 50 minutes, then a new class comes, then I have to focus on my 50 minutes and then the next 50 minutes and so on, that, I think you lose a bit of the overall…, of the students anyway. And then the school management is probably a more important factor there, making sure that there is daily physical activity for the students.	Being a teacher in middle school differs from elementary school<br><br>The teachers' primary job is to educate<br><br>Adult resources most be used properly<br><br>Management should make sure daily movement happens for the students	
Teacher: So, physical activity is really important.<br><br>(….)<br><br>Researcher: And how, how do you make this happen?<br><br>Teacher: One part to make it possible is by organizing the environment, I would like to have an outdoor exercise station like this outside, I would like to have a cross fit track…, I want a tractor tire out here that they [the students] can roll over. To get outside and get their heart rate up (…) So I think a lot is about the environment, making it possible.	Physical activity is perceived important (although not enacted)<br><br>There are many ideas about how to facilitate movement<br><br>'Effects' are wanted (e.g., high pulse)<br><br>Materials and physical spaces conditions movement (but takes time to change/is not in place yet)	*'I see no obstacles'*
Researcher: Mm, okay. Do you see any obstacles or opportunities for physical activities?<br><br>Teacher 1: Mm… Yes, so actually, it's the imagination that puts the stop to it really, you could involve some kind of movement into all lessons, some shorter movement break, uh, of like 5 minutes maybe, especially during longer lessons where they might have to follow some movement video or something. So, there are possibilities too, but the	Endless possibilities<br><br>Practicalities puts a stop to physical activities	

schedule is often…, uh…, a problem, if there is a short time between lessons. If you want outdoor activities, so to speak.<br><br>Researcher: Exactly. And why then, if you can just describe a little…?<br><br>Teacher 1: Uh… Yes, it becomes stressful to get from lesson to lesson if it is short. Time in between. Then they [the students] might not even have time to put on clothes, outerwear and such and do the activity and then they have to go to the next lesson. So, it takes a while…<br><br>Teacher 2: It takes time to get to the locker, hand in the books, take the other books and then you leave and you end up standing outside the classroom.		
Researcher: Yes, how do you make it [physical activity] happen, do you have any ideas?<br><br>Resource teacher: It is just about putting materials and conditions together, so that there is... Just putting students in a schoolyard and thinking that they will move around is probably quite unlikely. You need to see what they are interested in, arrange materials, make sure that staff are excited to go out with them.	Physical activities take systematic work<br><br>Materials conditions possibilities<br><br>Lack of pedagogic strategies conditions possibilities<br><br>Involve students and staff (cause now they are not)<br><br>Possibilities for movement need to be meaningful to students	
Researcher: Do you see any obstacles…?<br><br>Special education teacher: No, not really, but it’s probably more need of like an organization of those [staff] who are out and about [during recess]. Right now, we are just moving around [on the school premises].	No perceived obstacles for physical activity (although it is not happening)<br><br>Physical activities take systematic and organizational work	

Student: Today I'm going to the gym.<br>Researcher: Mm.<br>Student: I've been there a few times.<br>Researcher: Are you going there with a friend or your parents?<br>Student: I've been there with my parents but now I'm going with a friend.<br>Researcher: Mm. What kind of training is it?<br>Student: We will probably go to the gym, so...<br>Researcher: Okay, so strength training?<br>Student: Yes.	Some students have physical experience from during leisure time<br><br>Parents/social circle involved in leisure active lives	*It is not a level playing field*
'Many students here bring 'problems' with them right from the start, with learning, problems at home and so on. Then the focus of the lessons often becomes what needs to be learned instead of what is fun. It is unfair in a way, unfair between different schools and different students.' - Teacher<br>Field notes	Background matters in daily physical activities<br><br>There is no room for PA for certain schools/students	
PEH teacher: Yes, absolutely. Both me and the other PEH teacher at this middle school, we have sat and discussed a lot, and in some way want to run a [physical activity] project like this, because we see that the students, when we offer things, now we are going to play some football or, something extra, then it is the same students who come. And those are the students who do sports in their free time. And from a health perspective, those are not the ones we want to reach, I want to reach the ones that I have in the area like this, maybe girls with an immigrant background who have never played sports in their entire lives. No culture with them from home, have never done sports, those are the ones you want to reach so you can tell them/show what incredible health benefits you could get if you got them moving.	Background matters in physical education and health class<br><br>It is hard to reach the non-active students	

Researcher: What is it that you perceive as hard [with being physically active in school]?<br>Student 1: Um…, so I mainly like ’orienteering’ and then like, I'm not very fit. Um…, because I don't do any fitness sports like this, so it is kind of a problem when there is a lot like this where you have to run in a certain time.<br>(…)<br>Student 2: Yes, but I think it is like this, because if you go to sports like after school or something, then you have chosen something that is fun, that you are interested in, but at school it can be completely pointless things like running around in a circle for like 30 minutes, that is not that fun.	Students that find physical activities pointless<br><br>Physical activity is not rewarding (when you are not e.g., fit)	*Daily physical activities are (not) for everybody*
Researcher: Mm, okay. Have you played ping-pong?<br>Student: No. Well, it is mostly like this, I don't know, I like to play it, I usually do, we had to do it in sports but uh, I don't dare play with those ones [the boys].<br>Researcher: Okay. What, are they scary?<br>Student: No, no, well, it's just like these guys from the 8th grade and some from my class but I don't know, and there are usually never any girls who play.<br>Researcher: No, exactly. And then, then it is kind of a little bit to take that step and join in and play, it is pretty big then?<br>Student: Yes.<br>Researcher: Hmm. Why do you think there are so few girls?<br>Student: I don't know. Most of them just hang out with each other instead of doing anything.<br>Researcher: Okay, so that's kind of what you do during recess then?<br>Student: Yes.	Gender matters for access to physical activity<br><br>Age matter for access to physical activity<br><br>Intersection of gender and age	
Student: Yeah, I mean, I really don't like playing soccer or anything with the class. Because it feels like you kind of have to be good because otherwise people get mad. So, I hate playing soccer and volleyball and stuff like that.<br>Researcher: Are there any people who kind of get mad at you or like ah…<br>Student: Well, this school, I started here in the 7th grade, so we haven't had soccer or anything, we have just been outside now. But there hasn't been anything like this before,	Some are good at sports from before, some not<br><br>Hegemonic sporting cultures	

but at my old school back then, everyone, I mean everyone was doing it so that if you did something wrong then…, yeah, everyone got mad at you.<br><br>Researcher: Okay, that's right, yeah. Um… Then it kind of became a fight or like ah, what are you doing or…<br><br>Student: Yeah, exactly.	Students trying to avoid activities (e.g., soccer)	
‘That assignment [schools’ assignment to provide daily physical activity from students] is good for the school and important for the students to move’ (….) ‘I don’t want to look bad in front of the other students so that is why I don’t want to move’ - Student<br><br>Fieldnote	Student do not want to be perceived as bad in front of peers triumphs physical activity importance	
Teacher: I don't know. But even there you might be struggling a bit ‘against the wind’, and besides, if you are not the least bit interested in ball sports then it becomes a bit, well what do we have left, a bit too cool if you're in the 8th grade to jump rope maybe, or jump fence or do some bridge and scrimmage and all that stuff, it is probably more...	Interests/Activities are limited for middle school students due to social codes	
